# Efficacy of ultrasonic activation of NaOCl and orange oil in removing filling material from mesial canals of mandibular molars with and without isthmus

**DOI:** 10.1590/1678-775720150090

**Published:** 2016

**Authors:** Mirela Sangoi Barreto, Ricardo Abreu da Rosa, Manuela Favarin Santini, Bruno Cavalini Cavenago, Marco Antônio Húngaro Duarte, Carlos Alexandre Souza Bier, Marcos Vinícius Reis Só

**Affiliations:** 1 - Department of Stomatology, Federal University of Santa Maria, Santa Maria, RS, Brazil.; 2 - Department of Conservative Dentistry, Federal University of Rio Grande do Sul, Porto Alegre, RS, Brazil.; 3 - Department of Operative Dentistry, Endodontics and Dental Materials, Bauru School of Dentistry, University of São Paulo, Bauru, SP, Brazil.

**Keywords:** Micro-computed tomography, Endodontics, Retreatment, Ultrasonics

## Abstract

**Objectives:**

The aim of this study was to evaluate the volume of remaining filling material after passive ultrasonic irrigation (PUI) of sodium hypochlorite (NaOCl) and orange oil in mesial canals of mandibular molars, with and without isthmus.

**Material and Methods:**

Thirty mesial roots of mandibular molars were divided according to the presence or absence of isthmus. Canals were prepared and filled (Micro-CT #1). Filling was removed using rotary instruments, and specimens were sub-divided into three groups according to the irrigation procedures: Conventional – conventional irrigation with NaOCl, PUI/NaOCl – PUI of NaOCl (three activations, 20 seconds each), and PUI/orange oil – PUI of orange oil (Micro-CT#2). Specimens were enlarged using the X2 and X3 ProTaper Next instruments and submitted to the same irrigation protocols (Micro-CT #3).

**Results:**

No differences were found between the experimental groups in each stage of assessment (P>0.05). The volume of residual filling material was similar to those in Micro-CT #2 and Micro-CT #3, but lower than those observed in Micro-CT #1 (P<0.05). When groups were pooled according to the presence or absence of an isthmus, volume of residual filling material was higher in specimens presenting isthmus (P<0.05).

**Conclusions:**

PUI of NaOCl or orange oil did not improve filling removal. Isthmus consists in an anatomical obstacle that impairs the removal of filling material.

## INTRODUCTION

When endodontic failure occurs, nonsurgical endodontic retreatment is often indicated. Mesial root canals of mandibular molars are usually flattened mesiodistally, and sometimes contain an isthmus, which communicates the buccal with the lingual canals^22^. The inaccessibility of instruments and chemical irrigants into the isthmus impairs root canal disinfection^31^.

During root canal retreatment, filling material must be removed to enable re-preparation and disinfection of the root canal system^23^. The most commonly used techniques for filling removal involve hand files or rotary instruments^10^_._ These instruments can be used with chemical solvents to solubilize the gutta-percha filling^25^ ^,^ ^28^. However, due the complexity of the root canal system, complete removal of gutta-percha and sealer is difficult regardless of the employed technique^31^.

Organic solvents can be used during retreatment to facilitate root filling removal without damaging the tooth^26^. Chemical solvents present different proprieties that should be considered, such as efficacy in dissolving root filling material and cytotoxicity levels^3^. Orange oil is as effective as xylol in dissolving root canal filling^13^ ^,^ ^14^. Additionally, it has lower cytotoxicity when compared to eucalyptol, xylene, and chloroform^4^.

Several rotary systems were developed for root canal preparation^12^. ProTaper Next (Dentsply Maillefer, Ballaigues, Switzerland) is a new rotary system that must be used with continuous rotation. It consists of five instruments with variable taper: X1 (17/.04), X2 (25/.06), X3 (30/.075), X4 (40/.06), and X5 (50/.06). All of these instruments present a rectangular cross-section and must be passively inserted at the entire working length^17^. The ProTaper Retreatment kit (Dentsply Maillefer, Ballaigues, Switzerland) consists of three instruments designed specifically to remove root filling material from root canals. ProTaper Retreatment instruments have a convex triangular cross-section similar to that of the ProTaper Universal system (Dentsply Maillefer, Ballaigues, Switzerland).

To improve root canal disinfection, ultrasonic tips have been used in endodontics. Passive ultrasonic irrigation (PUI) has been used for removing root canal dressings, debris and the smear^6^ ^,^ ^29^. However, the effectiveness of PUI for filling removal has been poorly investigated.

Therefore, the aims of this study were: (1) to compare the volume of remaining filling material after conventional irrigation, PUI of NaOCl, and PUI of orange oil; and (2) to evaluate the influence of an isthmus in mesial root canals of mandibular molars on the remaining filling volume. The null hypotheses were: (1) there is no difference between the final irrigation protocols; and (2) the presence of an isthmus does not affect the volume of remaining filling material.

## MATERIAL AND METHODS

This study was approved by the Ethics Committee of the Federal University of Rio Grande do Sul. Thirty mesial roots of mandibular molars were used in this study. An initial micro-CT scan was used to select fifteen roots containing an isthmus and fifteen with independent mesiobuccal and mesiolingual canals using a desktop X-ray microfocus CT scanner (SkyScan 1174v2; Bruker-microCT, Kontich, Belgium). The scanning procedures were performed using the following parameters: 50 kV X-ray tube voltages, 800 μA anode current and voxel size of 14.4 μA. Only one specimen was scanned at a time. Scans with 1304x1024 pixels were obtained with acquisition intervals of 1° over a total of 360° rotation. Thus, data were recorded for tooth selection, and the sequences of scans were reconstructed using the NReconv1.6.4.8 (NRecon v.1.6.3; Bruker-microCT, Kontich, Belgium) software.

The exclusion criteria were: root fractures, presence of cracks, internal or external root resorptions, previous endodontic treatment, and an angle of curvature higher than 22°^21^. Crowns were removed using a double-sided diamond disc (Komet, Santo André, SP, Brazil) close to the cementoenamel junction. Canals were explored using a size 10 K-file (Dentsply Maillefer, Ballaigues, Switzerland). Working length (WL) was defined as 1 mm shorter than the distance from the cementoenamel junction to the apical foramen, where the file was just visible. Canal patency was obtained using #1, #2, and #3 PathFile instruments (Dentsply Maillefer, Ballaigues, Switzerland) and canals were prepared using S1, S2, and F1 ProTaper instruments (Dentsply Maillefer, Ballaigues, Switzerland) for all WLs. Irrigation procedures were performed using 2 mL of 2.5% sodium hypochlorite after each instrument change. After canal preparation, 5 mL of 17% ethylenediaminetetraacetic acid (Biodinâmica, Ibiporã, PR, Brazil) was used for three minutes. Finally, 2 mL of 2.5% sodium hypochlorite was used for the final rinse. All irrigation procedures were performed using silicon syringes (Ultradent Products Inc., South Jordan, UT, USA) and Endo-Eze tips (Ultradent Products Inc., South Jordan, UT, USA).

The root canal filling was placed using the single cone technique with an epoxy resin-based sealer AH Plus (Dentsply Maillefer, Ballaigues, Switzerland) and F1 gutta-percha cones (Dentsply Maillefer, Ballaigues, Switzerland). Endodontic sealer was mixed according to the manufacturer’s instructions and inserted into the canals using a Lentulo spiral (Dentsply Maillefer, Ballaigues, Switzerland). The canal opening was sealed with Cavit (3M ESPE, St Paul, MN, USA) and the specimens were stored at 100% humidity and 37°C for one week to allow the sealer to set. Then, the specimens were scanned to determine the initial volume of filling material (Micro-CT #1) as described above.

ProTaper Retreatment instruments (Dentsply Maillefer, Ballaigues, Switzerland) were used with 500 rpm and 2 N/cm of torque for removal of the filling as follows: the D1 file in the cervical area, the D2 file in the cervical and middle thirds, and the D3 file for all WLs. After each instrument change, root canals were irrigated with 2 mL of 2.5% NaOCl. After using the ProTaper Retreatment instruments, specimens were allocated into six groups according to the final irrigation procedures and the presence or absence of an isthmus (n=5):

- Conventional – Continuous irrigation with 10 mL of 2.5% NaOCl in each canal for 1 minute.

- PUI/NaOCl – Each canal was flushed with 0.5 mL of 2.5% NaOCl. Next, the solution was activated using passive ultrasonic irrigation with a size 20, 0.1 taper ultrasonic tip (Capelli e Fabris Ind., Santa Rosa do Viterbo, SP, Brazil), attached to a NAC Plus ultrasonic device (Adiel LTDA São Paulo, SP, Brazil). The ultrasonic tip was passively inserted into root canals 2 mm short of the WL. PUI was performed at 40 kHz for 20 seconds in each canal. The PUI protocol was repeated twice with 2 mL of 2.5% NaOCl *per* canal each time. Finally, continuous irrigation was performed with 6 mL of 2.5% NaOCl. The total volume of NaOCl was 10 mL in each root canal.

- PUI/orange oil – 0.5 mL of orange oil was used in each canal (Biodinâmica, Paraná, PR, Brazil). Next, the PUI protocol was performed as described above, using three activations of the solvent for 20 seconds in each root canal. Between activations, canals were flushed with 2 mL of 2.5% NaOCl and the solvent was refreshed. Finally, each canal was continuously irrigated with 6 mL of 2.5% NaOCl. Canals were dried with absorbent paper points and new micro-CT scans were performed (Micro-CT #2). In groups that received PUI, the movements of ultrasonic tip were performed toward the isthmus.

Canals were re-prepared with X2 and X3 ProTaper Next instruments (Dentsply Maillefer, Ballaigues, Switzerland) at all WLs with 300 rpm and 2 N/cm of torque. After using these instruments, specimens were flushed with 2 mL of 2.5% NaOCl. Finally, specimens were subjected to the same irrigation procedures previously described. Micro-CT #3 was performed to quantify the volume of the remaining filling material.

The amount of remaining filling material was volumetrically analyzed using the CTscan v1.11.10.0 (Brucker-microCT, Kontich, Belgium) software. The initial image was analyzed by limiting the region of interest (ROI) of the specimen with the CTscan software, and the new ROI data were saved in a separate folder. Then, the set of sample data was opened and the binary value was adjusted according to the raw images. The value was recorded and used as a parameter for Micro-CT #2 and Micro-CT #3. Finally, a 3D plug-in analysis was performed to quantitatively measure the amount of residual filling. This tool automatically calculates the overall volume (mm^3^) from the 3D image of binary selected objects (white color). For each sample, the volume was calculated at three root levels: 1 to 3 mm, 3 to 5 mm, and 5 to 7 mm from the apex. All measurements and analyses were performed by the same operator who was blinded from the irrigating protocols.

### Statistical analysis

Statistical analysis was performed using the SPSS 16.0 software (SPSS Co., Chicago, IL, USA). The Shapiro-Wilk test showed normal distribution of the data (P>0.05). Therefore, the volume of remaining filling material was analyzed using parametric tests. The repeated measures ANOVA and Tukey’s *post-hoc* tests were used to compare the volume of filling material in each experiment. The comparison of the irrigation protocols was performed using a one-factor ANOVA test. To assess if the presence or absence of an isthmus impairs the removal of the filling, Student’s *t*-test and the repeated measures ANOVA test were used. The level of significance was set to 5% for all statistical tests.

## RESULTS

**Figure 1:**
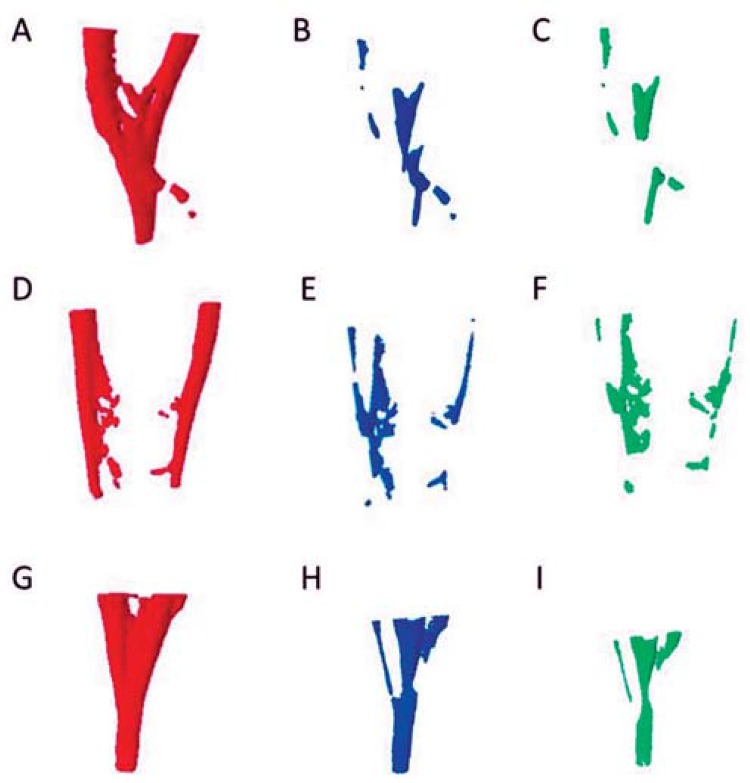
A, B and C represent specimens from the Conventional group; D, E and F from the PUI/NaOCl group; and G, H and I from the PUI/orange oil group. Images in red, blue and green indicate the volume of filling material at the baseline (Micro-CT #1), after filling removal and irrigation procedures (Micro-CT #2) and after re-preparation and irrigation procedures (Micro-CT #3), respectively

Initially, the volume of filling material in each experimental group was assessed. No differences were observed between *Conventional, PUI/NaOCl* and *PUI/orange oil* groups at any of the root levels analyzed (P>0.05). After Micro-CT #2, all groups showed a significant reduction of the residual filling volume (P<0.05). However, no differences between them were observed (P<0.05). When the specimens were re-prepared with ProTaper Next instruments and the irrigation protocols were repeated, the volume of remaining filling material was similar to those achieved after the first activation (P>0.05). After Micro-CT #3, no differences were observed between the irrigating protocols (P>0.05) (Table 1). Figure 1 shows representative images from *Conventional, PUI/NaOCl* and *PUI/orange oil* groups during the three experimental stages: Micro-CT #1 (initial), Micro-CT #2 (after filling removal and irrigation procedures) and Micro-CT #3 (after re-preparation and irrigation procedures).

**Table 1:** Mean and standard deviation of the volume (mm3) of filling material at baseline (Micro-CT #1), after filling removal and irrigation protocols (Micro-CT #2), and after re-preparation and irrigation protocols (Micro-CT #3)

	Baseline (Micro-CT #1)			
	1-3 mm	3-5 mm	5-7 mm	TOTAL
Conventional	0.71^A^	1.39^A^	1.81^A^	3.12^A^
	(±0.22)	(±0.36)	(±0.43)	(±1.21)
PUI/NaOCl	0.72^A^	1.32^A^	1.80^A^	3.20^A^
	(±0.25)	(±0.49)	(±0.56)	(±1.74)
PUI/orange oil	0.66^A^	1.07^A^	1.53^A^	3.25^A^
	(±0.21)	(±0.26)	(±0.29)	(±0.71)

	**Filling removal and irrigation procedures (Micro-CT #2)**			

	1-3 mm	3-5 mm	5-7 mm	TOTAL
Conventional	0.27^B^	0.50^B^	0.46^B^	1.23^B^
	(±0.18)	(±0.31)	(±0.34)	(±0.75)
PUI/NaOCl	0.36^B^	0.36^B^	0.37^B^	1.09^B^
	(±0.30)	(±0.38)	(±0.43)	(±1.05)
PUI/orange oil	0.23^B^	0.21^B^	0.26^B^	0.70^B^
	(±0.27)	(±0.23)	(±0.34)	(±0.81)

	**Re-preparation and irrigation procedures(Micro-CT#3)**			

	1-3 mm	3-5 mm	5-7 mm	TOTAL
Conventional	0.22^B^	0.47^B^	0.42^B^	1.12^B^
	(±0.18)	(±0.32)	(±0.32)	(±0.75)
PUI/NaOCl	0.27^B^	0.32^B^	0.34^B^	0.93^B^
	(±0.24)	(±0.38)	(±0.38)	(±0.95)
PUI/orange oil	0.17^B^	0.15^B^	0.23^B^	0.55^B^
	(±0.21)	(±0.17)	(±0.34)	(±0.68)

Different letters in the row, comparing similar root levels, indicate significant differences after repeated measures ANOVA and Tukey’s tests (P<0.05)

**Figure 2:**
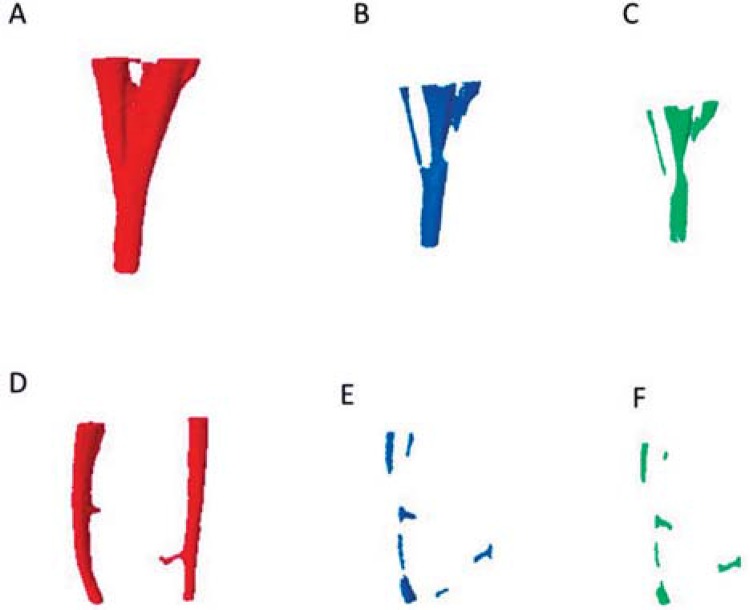
Images A, B and C represent the mesial root of a mandibular molar with the presence of an isthmus, and images D, E and F show the absence of an isthmus. Red denotes filling material at baseline (Micro-CT #1), blue is after filling removal with rotary instruments (Micro-CT #2); and green is after re-preparation with ProTaper Next (Micro-CT #3)

A larger volume of filling material was observed in specimens that contained an isthmus when compared to those that contained two independent canals (P<0.05) (Table 2). This pattern remained unchanged after using the ProTaper retreatment instruments and after re-preparation with ProTaper Next files (Figure 2). A smaller volume of filling material was observed after removal with rotary instruments (Micro-CT #2) and after re-preparation (Micro-CT #3) when compared to the volume at baseline (P<0.05), but no differences were observed between Micro-CT #2 and Micro-CT #3 (P>0.05).

**Table 2:** Mean and standard deviation of the volume of filling material at baseline, after filling removal with rotary instruments, and after re-preparation taking into consideration the presence or absence of an isthmus in the mesial root of mandibular molar

	Baseline (Micro-CT #1)			
	1-3 mm	3-5 mm	5-7 mm	TOTAL
Presence of an isthmus	0.83^Aa^	1.45^Aa^	1.91^Aa^	3.77^Aa^
	(±0.25)	(±0.40)	(±0.49)	(±1.39)
Absence of an isthmus	0.57^Ab^	1.07^Ab^	1.51^Ab^	2.62^Ab^
	(±0.12)	(±0.28)	(±0.28)	(±0.76)

	**Filling removal with rotary instruments (Micro-CT #2)**			

	1-3 mm	3-5 mm	5-7 mm	TOTAL
Presence of an isthmus	0.43^Ba^	0.57^Ba^	0.61^Ba^	1.63^Ba^
	(±0.26)	(±0.29)	(±0.35)	(±0.78)
Absence of an isthmus	0.13^Bb^	0.13^Bb^	0.13^Bb^	0.40^Bb^
	(±0.12)	(±0.19)	(±0.17)	(±0.43)

	**Re-preparation with ProTaper Next (Micro-CT #3)**			

	1-3 mm	3-5 mm	5-7 mm	TOTAL
Presence of an isthmus	0.34^Ba^	0.52^Ba^	0.57^Ba^	1.42^Ba^
	(±0.22)	(±0.32)	(±0.33)	(±0.73)
Absence of an isthmus	0.12^Bb^	0.10^Bb^	0.10^Bb^	0.32^Bb^
	(±0.12)	(±0.17)	(±0.17)	(±0.42)

Different uppercase letters in rows compare the presence or the absence of an isthmus in the same root level after each stage of retreatment using ANOVA and Tukey’s tests (P<0.05). Different lowercase letters in the columns compare the presence and the absence of an isthmus in each root level and stage of retreatment using Student’s t-test (P<0.05)

## DISCUSSION

The goal of root canal retreatment is to remove as much filling as possible to maximize root canal disinfection^11^. However, there is no technique capable of completely removing gutta-percha and endodontic sealer from root canal walls, especially when the canal is flattened^31^.

Some studies used invasive, destructive and two-dimensional methodologies to assess the amount of remaining filling material^24^. Só, et al*.* ^24^ (2008) sectioned the specimens longitudinally to assess the area of remaining filling material by using a dental operating microscope, which makes it impossible to evaluate one sample by different stages of retreatment. On the other hand, micro-CT is a quick, accurate and non-destructive method to assess filling removal^9^. Micro-CT enables the visualization of three-dimensional images with a high level of detail. However, the main advantage of micro-CT over other methodologies is that it allows for the acquisition of images of the same sample during different experimental stages^18^.

The aim of this study was to compare the volume of remaining filling material after conventional irrigation, PUI with NaOCl, and PUI with orange oil. Micro-CT #1 was performed to ensure that the volume of filling material at baseline was similar for all experimental groups. The results confirmed that the filling volumes in the *Conventional, PUI/NaOCl and PUI/orange oil* groups were not significantly different (P>0.05). Thus, the first null hypothesis was confirmed. Neither of the PUI protocols was capable of increasing the removal of filling material.

Cavenago, et al*.* ^7^ (2014) evaluated the volume of remaining filling material after retreatment with different procedures performed sequentially (i.e., mechanical removal, post-xylene and post-PUI). In contrast to the results of the present study, the authors found a smaller volume of filling material after PUI when compared to mechanical removal. It should be noted that those authors performed all retreatment procedures using an operating microscope with 5× magnification. Literature reports that using an operating microscope enhances the detection of retained filling material, especially at the cervical and middle thirds of mesial roots of mandibular molars^20^. At the apical portion, such visualization may be impaired due to the degree of curvature. This study also found residual filling material in all specimens, similar to many endodontic retreatment studies^7^ ^,^ ^12^.

The greatest reduction in filling material volume was observed after using ProTaper retreatment instruments and applying the irrigation protocols (Micro-CT #2) (P<0.05). Nevertheless, the mechanical action of the instruments was the main reason for the significant reduction in filling volume. On the other hand, neither *PUI/NaOCl* nor PUI/orange oil protocols were capable of reducing the volume of filling material within the root canal system after re-preparation using X2 and X3 ProTaper Next instruments (P>0.05). Even newly developed instruments are not able to contact all dentinal walls during a root canal preparation^30^. Additionally, Rödig, et al.^19^ (2014) observed residual filling material in all specimens after using the ProTaper Retreatment system, Reciproc instruments and Hedstroem hand files. The ProTaper Next system has a variable taper and rectangular cross-section, and uses an asymmetric rotary motion^17^. The combination of the rectangular cross-section with asymmetric rotary motion results in contact of only two cutting edges of the instrument with dentinal walls. The other two edges move freely, which reduces the torsion on the spirals and, thus, the risk of torsional fractures.

Several solvents have been evaluated for their efficacy in filling removal and toxicity levels^4^ ^,^ ^13^ ^,^ ^14^. Chloroform, xylene, eucalyptol and orange oil are the most commonly used solvents in endodontic retreatment. The main goal of combining solvents with PUI is to reach areas that of difficult access with endodontic instruments. Orange oil was chosen because of its low toxicity and for being able to dissolve gutta-percha, regardless of its concentration. According to Magalhães, et al.^13^ (2007) orange oil, eucalyptol and chloroform can similarly dissolve gutta-percha, but less so than xylene. On the other hand, Martos, et al.^14^ (2006) found that xylene and orange oil similarly dissolved gutta-percha and three endodontic sealers (i.e., Roekoseal, Endofill and Sealer 26). The main drawback reported concerning solvents is that they form a thin layer of softened gutta-percha and sealer that adheres to the canal walls and hinder its removal^8^.

In this study, *PUI/NaOCl* and *PUI/orange oil* were not able to reduce the amount of residual filling material (P>0.05). This result can be explained by the solvent contacting gutta-percha and forming a paste that adheres to the root canal walls by penetrating into canal irregularities. This impairs the removal of residual filling, especially within an isthmus. Table 2 shows the volume of filling material at baseline, after filling removal with rotary instruments, and after re-preparation in the presence and absence of an isthmus. Specimens with an isthmus contained a larger amount of residual filling material at all levels evaluated when compared to specimens with two independent canals (P<0.05). The penetration of gutta-percha and sealer into the isthmus and irregular areas makes removal of the filling more critical in roots with such anatomical features.

PUI appears to be effective for debris removal because of a phenomenon known as acoustic microstreaming and cavitation, which acts on root canals^1^. Castagna, et al.^6^ (2013) found that PUI used with 17% EDTA promoted greater debris removal, especially at the cervical third. In addition, the apical third was the most critical area that required cleaning. Some studies have evaluated the effectiveness of PUI on root canal dressing removal^2^ ^,^ ^5^. According to Capar, et al.^5^ (2014) PUI was the most effective protocol for calcium hydroxide removal from artificial standard grooves in the apical third. Corroborating these results, Ahmetoğlu , et al.^2^ (2013) observed that PUI was significantly more effective than Self-Adjusting Files and conventional irrigation for removing calcium hydroxide residue from the coronal, middle and apical thirds. Calcium hydroxide paste does not present adhesive properties, which facilitates its removal from the canal^6^. Epoxy resin-based sealers, in contrast, present good mechanical properties and cohesive resistance, which make more difficult to detach the endodontic sealer from the dentin wall when using PUI^27^. These factors may explain the presence of sealer in the canal walls and anfractuosities after endodontic retreatment.

Removal of the filling material and re-preparation of narrow and curved canals presents a challenge for clinicians^19^. When centering rotary instruments during re-preparation of the canal it is common to leave unprepared areas within the isthmus and flattened canals^12^. Because of this, mesial roots of mandibular molars were included in this study. In addition, these roots have an isthmus in 55% of cases^15^. Therefore, 50% of the samples studied were mesial roots with an isthmus and 50% contained independent mesio-buccal and mesio-lingual canals. Because no difference was observed among the irrigation protocols, data were pooled to assess the influence of the presence of an isthmus on the residual volume of filling material.

This study model contains some limitations. All procedures were performed in a controlled environment with extracted and crownless teeth that may not be equivalent to an average clinic situation. Additionally, an operating clinical microscope was not used. According to Pécora and Andreana^16^ (1993), the use of an operating clinical microscope enhances the visualization of root canal anatomy and improves sanitization and root canal preparation procedures.

## CONCLUSIONS

Given the findings of this study and the limitations described, it can be concluded that passive ultrasonic activation of NaOCl and orange oil did not improve the removal of filling material from mesial roots of mandibular molars when compared to conventional irrigation with NaOCl. Additionally, the isthmus is an anatomical obstacle that impairs the removal of filling material from mesial root canals of mandibular molars.
